# Acute Rheumatic Fever Diagnostic Network (ARC Network) clinical recruitment protocol

**DOI:** 10.1136/bmjopen-2025-114968

**Published:** 2026-03-25

**Authors:** Ndate Fall, Asha Clare Bowen, Samantha Buonfiglio, Joshua Reginald Francis, Christopher Gorman, Renata Fonseca Mendoza, Ela Fernandes, Nicole J Moreland, Maria Carmo Pereira Nunes, Craig Sable, Masood Sadiq, Casey P Shannon, Tom Parks, Anna Ralph, Amy Sanyahumbi, Rachel Sarnacki, Scott Tebbutt, Rachel Webb, Jonathan Carapetis, Andrea Zawacki Beaton, ARC Diagnostic Network

**Affiliations:** 1 Heart Institute, Cincinnati Children’s Hospital Medical Center Burnet Campus, Cincinnati, Ohio, USA; 2 The Kids Research Institute Australia, Nedlands, Western Australia, Australia; 3 Global and Tropical Health Division, Menzies School of Health Research, Darwin, Northern Territory, Australia; 4 Paediatrics, Royal Darwin Hospital, Darwin, Northern Territory, Australia; 5 Children’s State Hospital, Fiera De Santana, Bahia, Brazil; 6 Menzies School of Health Research Timor-Leste, Díli, Timor-Leste; 7 Molecular Medicine and Pathology, The University of Auckland Faculty of Medical and Health Sciences, Auckland, New Zealand; 8 Univ Fed Minas Gerais, Belo Horizonte, Brazil; 9 Ochsner Health, New Orleans, Louisiana, USA; 10 Paediatric Cardiology, The Children’s Hospital/ICH, Lahore, Punjab, Pakistan; 11 University of British Columbia, Vancouver, British Columbia, Canada; 12 St Paul’s Hospital, Vancouver, British Columbia, Canada; 13 Imperial College London, London, UK; 14 Menzies School of Health Research, Charles Darwin University, Darwin, UK; 15 Texas Children’s Hospital, Houston, Texas, USA; 16 Baylor foundation Malawi, Lilongwe, Malawi; 17 Cardiology, Children’s National Medical Center, Washington, District of Columbia, USA; 18 The University of Auckland Faculty of Medical and Health Sciences, Auckland, New Zealand; 19 Starship Children’s Hospital, Auckland, New Zealand; 20 University of Western Australia, Crawley, Western Australia, Australia; 21 Child and Adolescent Health Service, Nedlands, Western Australia, Australia; 22 Cardiology, Cincinnati Children’s Hospital Medical Center, Cincinnati, Ohio, USA; 23 ARC Diagnostic Network, Cincinnati, Ohio, USA

**Keywords:** Paediatric cardiology, Valvular heart disease, Cardiovascular Disease, Public health

## Abstract

**Abstract:**

**Introduction:**

Rheumatic heart disease, a major cause of morbidity and mortality in low- and middle-income countries, results from acute rheumatic fever (ARF), for which no diagnostic test currently exists. The ARF Diagnosis Collaborative Network (ARC Network) was established to address this gap by recruiting a rigorously phenotyped, globally representative cohort of children and adolescents with ARF and controls to support biomarker discovery. This paper describes the ARC Network’s multicentre recruitment strategy and standardised procedures for classifying ARF cases and controls, enabling robust development of novel, accessible diagnostics.

**Methods and analysis:**

The ARC Network recruits children and adolescents aged 3–18 years with suspected ARF and matched controls across four countries. Clinical, laboratory and echocardiographic data are collected using standardised protocols. Echocardiographic and clinical core teams provide rigorous standardised case review. Biospecimens are processed locally following harmonised procedures and shipped to a central biobank for long-term storage and future biomarker studies. Centralised training, quality control and a research database ensure high-quality, globally representative data to support ARF biomarker discovery.

**Ethics and dissemination:**

The ARC Network follows Institutional Review Board protocols with local ethical approval and informed consent, and oversees clinical data and biobank use for ARF research.

**STRENGTHS AND LIMITATIONS OF THIS STUDY**

- Multicountry recruitment of acute rheumatic fever (ARF) cases and controls, representing four continents to ensure geographical diversity and representation of ARF-endemic populations.
- Centralised training, standard protocols, rigorous case adjudication support high-quality, harmonised data and biospecimen collection.
- Will be the largest and among the only contemporary collections of ARF samples, supporting the work of the ARC Network but also future research in a globally neglected field.
- Limited access to commercially available anti-streptococcal antibody profiling necessitates centralised assays and highlights the challenge of diagnosis for ARF globally.

## Introduction

 Rheumatic heart disease (RHD) is an acquired cardiovascular condition affecting an estimated 55 million people worldwide and remains a leading cause of morbidity and premature mortality in low- and middle-income countries.^1^ RHD is the long-term consequence of acute rheumatic fever (ARF), an autoimmune complication that can occur in some individuals following superficial infection with *Streptococcus pyogenes* (group A *Streptococcus* or GAS).^2^

Despite decades of research, the absence of a definitive diagnostic test for ARF remains a major barrier to effective prevention of RHD. Gold standard for diagnosis is the Jones Criteria, which relies on a complex integration of clinical signs, laboratory markers and imaging findings. Even in well-resourced settings, this approach can be challenging due to the heterogeneous and overlapping presentation of ARF. In low- and middle-income settings, these diagnostic challenges are further amplified by limited access to laboratory testing, echocardiography and specialist expertise.^3 4^ The development of improved, accessible diagnostic tools for ARF is a critical step towards reducing the global burden of RHD.

The Acute Rheumatic Fever Diagnosis Collaborative Network (ARC) was established to address this diagnostic gap. The ARC Network brings together global expertise in immunology, microbiology, infectious diseases, clinical cardiology, echocardiography, telemedicine and data science to identify and validate biomarkers that could support the development of a novel, accurate and widely deployable diagnostic test for ARF.

At the core of The ARC Network’s biomarker discovery strategy is the recruitment of a rigorously phenotyped, geographically diverse cohort of children and adolescents with confirmed ARF, along with a set of predefined control groups. The careful selection and characterisation of cases and controls is essential for candidate biomarker discovery and validation. Moreover, global representation is critical to ensuring that any future diagnostic test for ARF is generalisable across diverse populations and health system settings.

However, identifying and enrolling ARF cases presents unique challenges, especially in low-resource settings where diagnostic infrastructure is limited and disease presentation may be delayed or missed. Additionally, the presentation of ARF can be subtle and overlapping with other infectious and inflammatory diseases, leading to misclassification. To overcome these barriers, The ARC Network has implemented a standardised recruitment and classification protocol across four continents, supported by remote mentorship, image sharing and centralised case adjudication. This coordinated, multicentre approach enables the generation of high-quality, harmonised data essential for robust biomarker discovery and diagnostic development.

In this paper, we describe The ARC Network’s clinical recruitment network and outline the operational definitions and procedures used to classify ARF cases and controls across sites.

## Methods and analysis

The overall objective of the ARC Network is to identify candidate biomarkers that could be translated into an ARF diagnostic fit for purpose in low- and middle-income country settings. In four ARC recruitment sites, patients with ARF and control patients are enrolled with blood samples collected for biomarker discovery.

**Figure 1:**
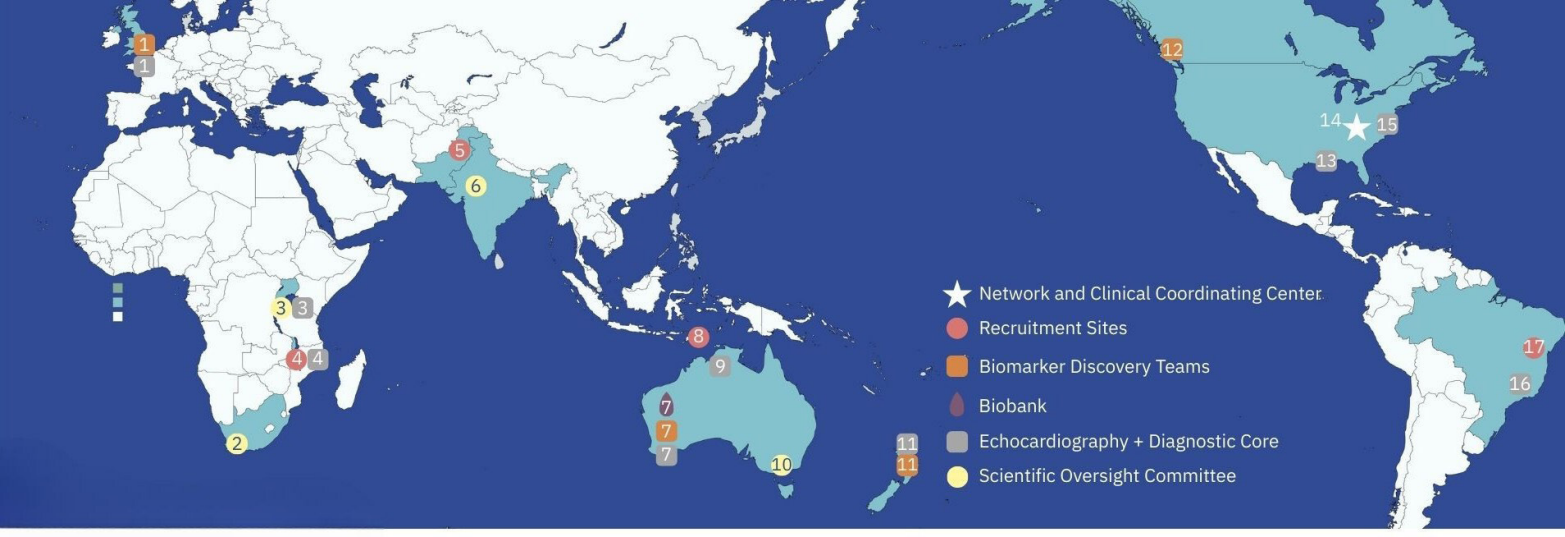
Participating sites. This map includes the Clinical Coordinating Center (CCC), Members of the Echocardiography (EC) and Diagnostic Cores (DC), Biobank (BB), Biomarker Discovery Teams (BD), Recruitment Sites (RS) and Scientific Oversight Committee Members (SOC). Institutions with role designation running from left to right on the above map: (1) Imperial College in partnership with Sanquin Labs, London England+Amsterdam Netherlands (DC, BD); (2) Medical Research Council, Cape Town South Africa (SOC); (3) Uganda Heart Institute and the Rheumatic Heart Disease Research Collaborative in Uganda (EC, DC, SOC); (4) Kamuzu Central Hospital partnering with Baylor College of Medicine and the University of North Carolina, Lilongwe, Malawi (EC, RC); (5) The Children’s Hospital and Institute of Child Health, Lahore, Pakistan (RC); (6) All India Institute of Medical Sciences, New Delhi, India (SOC); (7) The Kids Research Institute+Biotome, Perth, Australia (DC, BB, BD); (8) Guido Valadares National, Dili, Timor-Leste (RC); (9) Menzies School of Health Research, Darwin, Australia (DC, RC site support); (10) Murdoch Children’s Research Institute+The University of Melbourne+Hudson Institute of Medical Research, Melbourne, Australia (SOC); (11) University of Auckland, Auckland, New Zealand (DC, BD); (12) The Proof Center, Vancouver, British Columbia, Canada (BD); (13) Ochsner Medical Center, New Orleans, Louisiana, USA; (14) Cincinnati Children’s Hospital Medical Center, Cincinnati, Ohio, USA (CCC); (15) Children’s National Medical Center, Washington, DC, USA (EC); (16) Universidade Federal de Minas Gerais, Belo Horizonte, Brazil (EC); (17) Hospital Estadual da Criança, Bahia, Brazil (RS).

The ARC Network is organised around two synergistic workstreams: one focused on clinical recruitment and biobanking, and the other on biomarker discovery and validation. While biomarker discovery is a key downstream aim of the network, this manuscript focuses on the clinical recruitment infrastructure that underpins this work. In total, 13 countries participate in the network (map, figure 1).

The clinical work package is led by clinicians and investigators based both within and outside ARF-endemic regions with expertise in the recruitment and phenotypic classification of ARF cases and controls. This effort is coordinated through a centralised Clinical Coordinating Center and supported by a set of specialised teams that ensure standardisation, quality and rigour across all participating sites.

**Figure 2:**
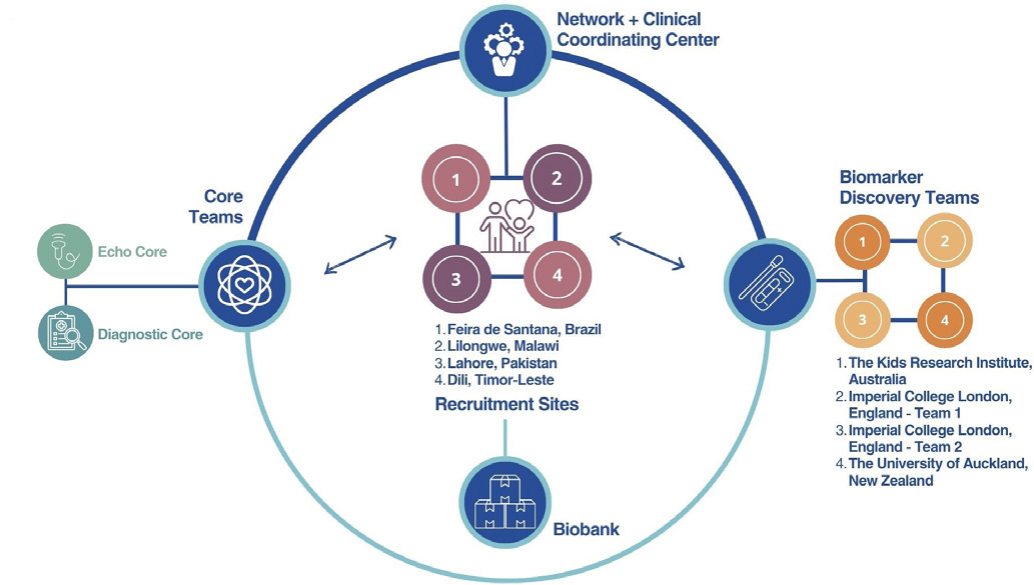
Acute Rheumatic Fever Diagnosis Collaborative Network organisation and teams. These teams work together to execute global recruitment and to deliver samples to the biomarker discovery teams that are looking for a new diagnostic test for acute rheumatic fever.

The sections that follow describe the structure and function of the Clinical Coordinating Center, recruitment sites, Echocardiography Core team, Clinical Adjudication Core team, Biobank and the Scientific Oversight Committee. Figure 2 illustrates the overall organisation of the ARC Network.

### Clinical Coordinating Center

The Clinical Coordinating Center, based at Cincinnati Children’s Hospital, serves as the operational hub of the ARC Network. It oversees the day-to-day activities and ensures alignment and consistency across all participating sites. As the anchor of network operations, the centre delivers comprehensive protocol training, manages data quality and integrity and provides regulatory guidance to participating teams.

The coordinating centre works in close partnership with local principal investigators and site leadership teams who play a central role in recruitment oversight, contextual adaptation of study procedures and implementation within their respective health systems. While governance is centralised to ensure harmonisation, operation decisions are informed by local expertise and site-specific conditions.

The coordinating centre also maintains the centralised Research Electronic Data Capture (REDCap) database^5 6^ used for clinical data collection and plays a central role in harmonising recruitment strategies, case adjudication processes and follow-up procedures. This centralised governance model ensures that clinical data are collected, cleaned and managed in a standardised manner, supporting cohesive, high-quality research.

#### Recruitment sites

The foundation of the ARC global cohort rests on four international recruitment sites including: (1) Hospital Estadual da Criança in Feira de Santana, Bahia, Brazil; (2) The Children’s Hospital and Institute of Child Health, Lahore, Pakistan; (3) Kamuzu Central Hospital and surrounding health centres, partnered with Baylor College of Medicine and UNC Project in Lilongwe, Malawi; and (4) Guido Valadraes National Hospital in Dili, partnered with Menzies School of Health Research, Timor-Leste . All of these facilities are national or tertiary-level referral hospitals. Each works through its network of referring providers and clinics to increase recruitment of ARF from the community as well as recruiting from the inpatient wards.

These sites were selected for geographical diversity and high incidence of ARF and RHD. They identify and enrol children and adolescents between the ages of 3 and 18 years with suspected ARF as well as control participants across four predefined categories: children with chronic RHD, those with superficial GAS infections but no ARF, individuals with other clinically overlapping infectious or inflammatory conditions, and healthy controls.

#### Echocardiography Core

The Virtual Echocardiography Core, based at Children’s National Hospital (Washington DC, USA) and Ochsner Children’s Hospital (New Orleans, Los Angeles, USA) plays a central role in standardising cardiac image acquisition and interpretation across the ARC Network. This core includes five cardiologists from the USA, Brazil and Uganda.

Recruitment sites are equipped with handheld echocardiography devices (Philips Lumify, Netherlands) and have access to a cloud server (Tricefy, Trice Imaging, Miami, Florida) for image upload, storage and interpretation. Sites receive comprehensive training from the Echocardiography Core team to ensure adherence to a standardised 10-view imaging protocol.

#### Clinical Diagnostic Core

The Clinical Diagnostic Core, based at The Kids Research Institute (The Kids) in Perth, Australia, oversees the review of all clinical, laboratory and imaging data and conducts final diagnostic adjudication. The core includes five standing members: paediatric infectious disease physicians from the UK, Australia, New Zealand and a paediatric cardiologist from Uganda.

#### Biobank

The ARC Biobank, housed at The Kids in Perth, Australia, supports the collection, processing, shipment and storage of all biospecimens collected through the ARC Network. The biobank trains site teams in standardised protocols for collecting and processing whole blood, serum, plasma and buffy coat and provides the necessary consumables to ensure standardisation across sites.

One site—the UNC-Project lab in Malawi—also collects peripheral blood mononuclear cells, which is not feasible at other sites due to infrastructure limitations. Specimens are shipped quarterly under strict cold chain conditions and tracked using a centralised inventory system.

#### Biomarker discovery

The biomarker discovery teams span research laboratories at The Kids Research Institute in Perth, Australia, the University of Auckland in New Zealand and Imperial College in London. Discovery platforms include antigen arrays, proteomics, RNA sequencing and profiling of soluble immune factors.

#### Scientific oversight committee

The ARC Network is guided by an independent Scientific Oversight Committee (SOC), comprising internationally recognised experts in ARF, infectious diseases, clinical research, cardiology, immunology, epidemiology and vaccine and diagnostic development. The committee includes members with extensive experience working in ARF-endemic regions and representation from low- and middle-income settings. The SOC provides critical external review of scientific progress and offers strategic recommendations at key decision points. Their leadership ensures scientific rigour, integrity and alignment with the ARC Network’s mission to advance the diagnosis and understanding of ARF.

A complete list of ARC Network investigators, site investigators and core members is provided in online supplemental table S1.

### Recruitment

#### Site selection

A structured pre-selection process was conducted to identify recruitment sites that demonstrated geographic diversity, endemic burden of RHD, strong interest in ARF/RHD research and capacity for research and infrastructure development.

#### Provision of supplies

The ARC Network provides all recruitment sites with standardised supplies essential for study procedures. These include handheld echocardiography devices, blood collection kits, reagents and sample processing materials such as centrifuge tubes, cryovials and barcoded labels. Each site is equipped with temperature-monitored freezers to maintain proper biospecimen storage −80 °C.

The biobank oversees the ongoing coordination of supply replenishment and manages the complex logistics of international sample shipments and approvals. This centralised management from the biobank ensures the integrity and consistency of biospecimen collection across the network.

#### Initial and ongoing training

All study personnel participate in a centralised, comprehensive training programme designed to promote consistent implementation of study procedures across ARC Network recruitment sites. At study launch, staff from the Clinical Coordinating Center and the Biobank Core travel to each site to deliver in-person training including regulatory compliance, documentation, informed consent procedures, data collection, electronic (REDCap) data entry and biospecimen collection, processing and storage.

The Echocardiography Core conducts virtual training sessions on cardiac image acquisition and sharing. Training is tailored to local contexts, documented and tracked to support ongoing compliance.

To maintain high standards and provide ongoing support, the Clinical Coordinating Center has established multiple channels for continuous engagement. Dedicated WhatsApp groups facilitate real-time communication and enable rapid resolution of site-level questions. Quarterly data digests summarise database updates, frequently asked questions and key operational reminders. A monthly recruitment dashboard tracks enrolment progress against targets. Refresher training on data entry procedure is conducted as needed, and quarterly remote monitoring ensures sustained protocol adherence.

#### Recruitment materials

Each recruitment site collaborates with the Clinical Coordinating Center to develop culturally and contextually appropriate outreach materials that promote awareness and encourage early presentation of suspected ARF cases. Using a general template provided by the coordinating centre, site teams adapt materials—including posters, flyers, symptom checklists and radio scripts—to reflect local language, health literacy levels and cultural norms.

Materials are further tailored to the specific type of health facility involved in recruitment—whether outpatient clinics, hospitals or community health centres—to ensure relevance and accessibility. Outreach efforts extend beyond clinical settings, with materials distributed in hospitals, clinics and community spaces to engage families where they live and seek care.

**Figure 3:**
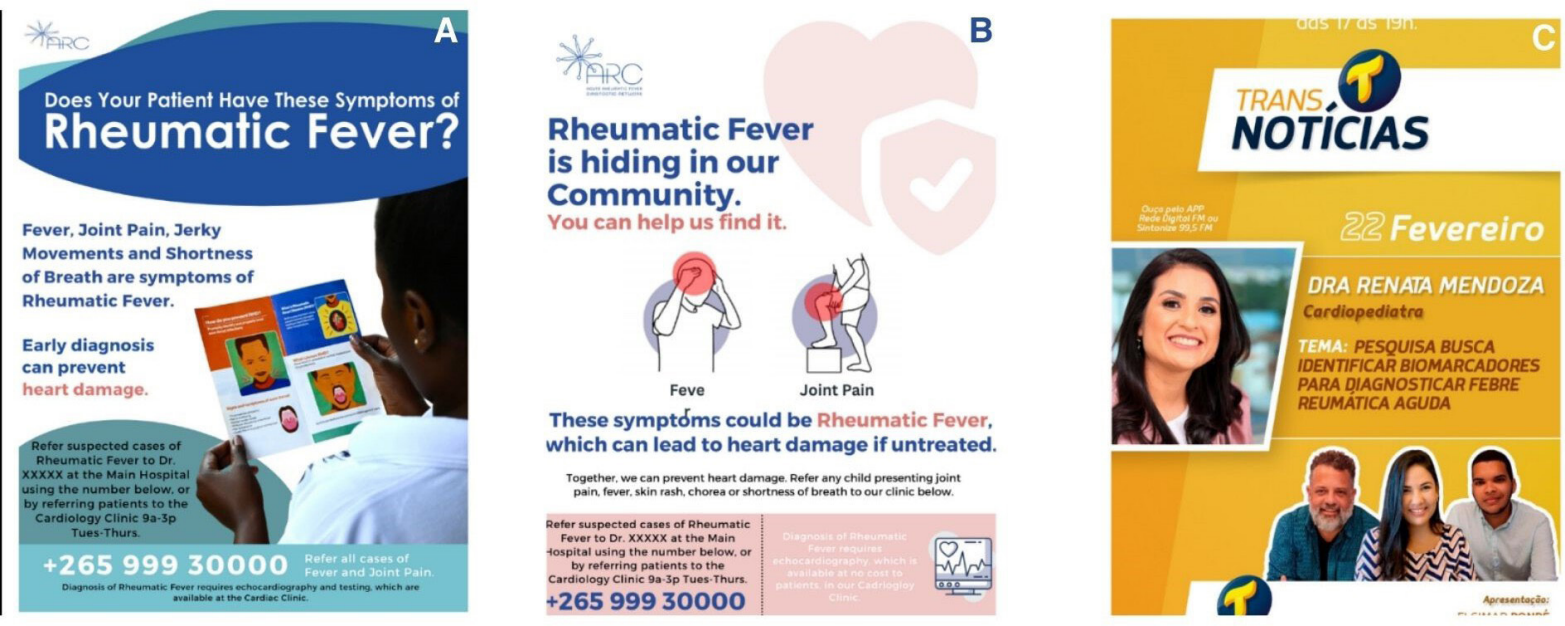
Examples of Acute Rheumatic Fever Diagnosis Collaborative Network recruitment materials. (A) Provider-facing poster used in Malawi. (B) Community-facing poster used in Malawi. (C) Poster to advertise a radio show used in Brazil.

Figure 3 shows examples of recruitment materials developed and adapted across sites, illustrating culturally tailored approaches to community engagement and case identification.

#### Recruitment categories

There are five recruitment categories. Each recruitment site aims to enrol at least 50 participants in each of the categories for a minimum recruitment of 250 participants. The ARC Network employs a phased biomarker discovery and validation strategy aligned with a PRoBE (Prospective Specimen Collection with Retrospective Blinded Evaluation) design to enhance methodological rigour and reproducibility in biomarker research.^7^ In the initial recruitment phase, a target of ≥250 participants (≥50 per recruitment category) was selected to ensure adequate phenotypic representation across recruitment strata while balancing feasibility across four endemic sites and preserving geographical diversity. This cohort contributes to a larger planned validation dataset (n ≈1000; approximately 200 cases and 800 controls), which is powered to support robust clinical prediction modelling. Based on established guidance for multivariable model development and shrinkage considerations,^8^ this sample size permits evaluation of models of increasing complexity while limiting overfitting (<10% coefficient shrinkage) and allowing precise estimation of discriminative performance (±0.05 area under the curve (AUC)) for models targeting AUC thresholds ≥0.80–0.90 with up to 20–40 parameters.

- **Suspected ARF**: Children and adolescents aged 3–18 years who present with one or more of the following: joint pain, suspected carditis, suspected chorea or known RHD with concern for recurrent ARF.
- **RHD controls**: Children and adolescents aged 3–18 years with RHD confirmed by echocardiography without acute signs/symptoms of ARF or other infectious or inflammatory conditions.
- **Superficial GAS**: Children and adolescents aged 3–18 years with suspected superficial GAS infection of the throat (pharyngitis) or skin.
- **Overlapping conditions**: Children and adolescents aged 3–18 years with other known infectious or inflammatory conditions that have some clinical overlap with ARF (fever, joint pain, rash, movement disorder, cardiac symptoms) but do not have ARF.
- **Healthy controls**: Asymptomatic children and adolescents aged 3–18 years with no recent illness or family history of ARF.

### Data collection

#### Clinical and epidemiological data

Standardised data collection instruments are used at the enrolment visit to collect data on demographics, history of presenting illness, medical history and family history. Each participant undergoes a standardised focused clinical examination by a trained provider. This examination includes photographs if there are skin or joint findings and a short video if there is suspected chorea.

A risk factor assessment and an assessment of quality of life are undertaken, with more details provided below. All children receive standard laboratory and cardiac testing. Participants enrolled under the category of suspected ARF are invited to have additional follow-up visits at 1, 3, 6 and 12 months. At these visits, they undergo a follow-up history, clinical examination, cardiac testing, as well as laboratory evaluation at 3 months.

#### Risk factors

Elements of the risk factor assessment were selected based on an extensive literature review.^9^ Evaluation included a measure of crowding according to the Argentinian National Institute of Statistics and Census (INDEC).^10^ Socioeconomic status was assessed using the Water, Assets, Maternal Education and Income Index,^11^ designed for discriminating between socioeconomic status in very-low resource environments. Oral health, access to primary care services and nutrition evaluation, and an ARF knowledge assessment were also collected.^9^ The Paediatric Quality of Life Inventory Generic Core Scales^12^ were selected due to their extensive cross-cultural validation, having been translated into more than 60 languages and demonstrating reliable and valid psychometric properties across diverse international paediatric populations^13^; however, formal local validation was not conducted within the four participating study countries and will be acknowledged as a limitation in subsequent analyses.

#### Cardiac testing

Each participant undergoes a standard 12-lead ECG and a focused echocardiogram at enrolment and each follow-up visit (for those with suspected ARF). ECGs are uploaded in PDF format to the REDCap database and read by a single paediatric cardiologist member of the diagnostic core.

Echocardiograms are performed using Philips Lumify (Netherlands) handheld ultrasound devices paired with secure Windows-based tablets, deployed at each site during early capacity-building visits. A simplified, standardised 10-step imaging protocol guides image acquisition in two-dimensional and colour Doppler, targeting key left-sided cardiac structures and is designed to be completed in under 15 min.

All echocardiograms are saved in the digital imaging and communication in medicine (DICOM) format and uploaded daily to a secure cloud server (Tricefy, Trice Imaging, Miami, Florida). The Echocardiography Core staff provide ongoing technical support and weekly feedback to sites, confirming study receipt and offering image quality assessments. Quality control is maintained through centralised review of each scan’s completeness and diagnostic quality, with a goal of ≥80% protocol adherence. Sites receive regular performance updates and additional training is offered as needed to ensure imaging meets standards required for adjudication.

#### Laboratory testing

Standard laboratory assessments include complete blood count, erythrocyte sedimentation rate, C-reactive protein (CRP), antistreptolysin O (ASO) titre and GAS PCR from throat or skin swabs as indicated. Additional testing is performed as clinically warranted. Due to local capacity constraints and lack of commercially available assay kits, anti-deoxyribonuclease B (ADB) testing cannot be performed at the recruitment sites. Serum aliquots are shipped to Auckland, New Zealand, where ASO titres and CRP are repeated and ADB titres are performed in an accredited clinical laboratory (Middlemore Hospital Laboratory).

#### Sample collection, processing and storage for the ARC Biobank

Biospecimens for storage are collected, processed and stored at the ARC Network sites using a standardised protocol to ensure consistency and quality.^14 15^ At enrolment, trained staff collect blood into pre-labelled vacutainers (serum-separating tube, ethylenediaminetetraacetic acid containing tube and PAXgene tube) in a defined order. Samples are processed into aliquots and stored in monitored freezers before quarterly, International Air Transportation Association-compliant shipments to the central ARC Biobank. At the biobank, samples are logged and stored long-term at −80°C in temperature monitored freezers.^16 17^

### Data quality

**Figure 4:**
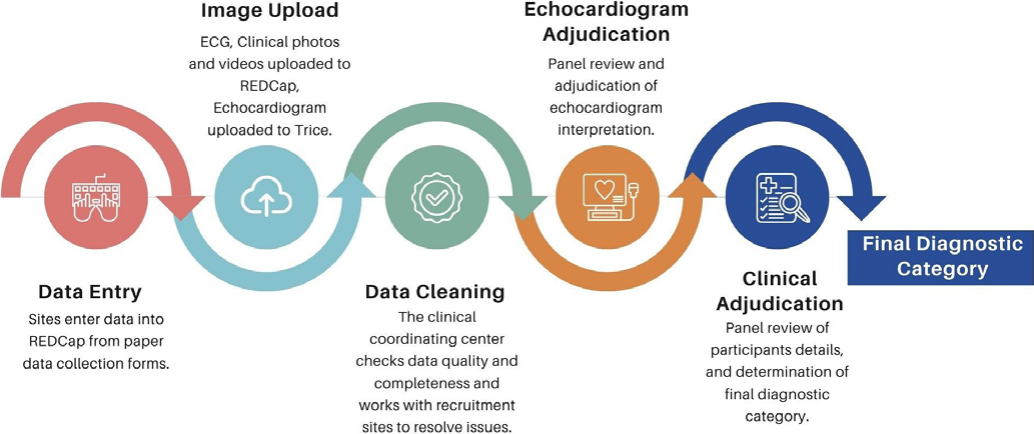
The flow of data in Acute Rheumatic Fever Diagnosis Collaborative Network from data entry at recruitment sites to final classification. A multistage process ensures consistent and rigorous participant classification using the expertise of team members around the globe. REDCap, Research Electronic Data Capture.

All data undergo systematic cleaning to ensure accuracy and consistency across sites. The Clinical Coordinating Center conducts regular audits to identify missing data, out-of-range values and logical inconsistencies. Discrepancies, such as laboratory values outside of the known range, are flagged through automated checks and resolved with site teams. All corrections are tracked to maintain an audit trail. Records are reviewed to confirm completeness before release to the adjudication teams. Figure 4 shows the flow from data collection to final adjudication.

### Diagnostic adjudication

#### Echocardiogram adjudication

The Echocardiography Core reviews each echocardiographic study for quality and completeness before adjudication. Each echocardiographic study is randomly assigned to two cardiologist members of the Echocardiography Core, using a predetermined case assignment algorithm. Reviewers complete these assessments blinded to others’ interpretation and to all clinical data.

Echocardiogram results are classified by the 2023 World Heart Federation criteria.^18^ Discrepancies are resolved through live virtual consensus review. Studies are categorised as normal, meets criteria for ARF/RHD (mild or moderate/severe), congenital heart disease, cardiomyopathy, with additional space for diagnostic comments.

The core captures the presence and severity of pericardial effusion and of reduced left ventricular function. The reviewing panel also documents the type, severity and distribution of valvular lesions. Serial studies are assessed for disease progression or regression.

#### Clinical adjudication process

After echocardiogram adjudication, each participant file is randomly assigned to two independent clinical reviewers via a predefined distribution list. Adjudication follows structured diagnostic algorithms developed by the ARC Diagnostic Core. Each participant’s data is independently reviewed by two of these ARF experts with reference to the 2015 Jones Criteria^4^ and thresholds for streptococcal serology suited to high-risk populations.^19 20^ When discrepancies arise, a structured consensus process is conducted through virtual or in-person case review meetings to reach a diagnostic classification. All classifications also undergo algorithmic review using a custom script written in R software (V.4.4.2). After this, cases with outstanding discrepancies are returned to the adjudication panel, and if a final classification cannot be reached, the participant is excluded from the analysis. This rigorous, transparent approach ensures consistent and accurate case definitions across the cohort, with feedback provided to the recruitment sites.

#### Diagnostic definitions

The ARC adjudication framework includes a detailed set of diagnostic categories to capture the clinical complexity of ARF and related conditions. Cases meeting the 2015 Jones Criteria^4^ are classified as definite ARF, further subcategorised as likely first episode, first episode with suspected underlying RHD or recurrence.

Alternate diagnoses include confirmed RHD (with or without active inflammation), overlapping conditions (eg, congenital heart disease, other infections) and specific diagnoses such as acute post-streptococcal glomerulonephritis or superficial GAS infections. A comprehensive list of non-GAS known alternate diagnosis subcategories is provided in online supplemental table S2. Additional categories include history of ARF or RHD with normal echocardiograms, possible ARF (minor criteria with high suspicion) and provisional categories like unknown alternate diagnosis or not enough evidence pending further data.

This classification system enables consistent, evidence-based adjudication across a diverse global cohort. Full category definitions are listed in table 1

**Table 1:** Acute Rheumatic Fever Diagnosis Collaborative Network diagnostic definitions

Category	Definition
First documented ARF episode and likely first episode of ARF	Meets criteria for definite ARF as outlined in the 2015 Jones Criteria.^4^ No known history of prior ARF or RHD, echocardiogram suggestive of first episode ARF (echocardiogram: most consistent with ARF or equally consistent with ARF/RHD at the adjudication panel’s recommendation).
First documented ARF episode with concern for likely past ARF/RHD	Meets criteria for definite ARF as outlined in the 2015 Jones Criteria.^4^ No known history of prior ARF or RHD, echocardiogram most consistent with underlying RHD (echocardiogram: most consistent with RHD at the adjudication panel’s recommendation).
Definite ARF, recurrence	Meets criteria for definite ARF as outlined in the 2015 Jones Criteria.^4^ Known history of prior ARF or RHD.
Known alternate diagnosis, RHD, healthy	Known history of RHD confirmed by echocardiographic evaluation (echo: most consistent with RHD, or equally consistent with ARF/RHD). Or new diagnosis of RHD on echocardiogram (same echocardiogram findings). No evidence of inflammation on baseline laboratories (ESR <30 and CRP <3).
Known alternate diagnosis, RHD, unhealthy	Known history of RHD confirmed by echocardiographic evaluation (echo: most consistent with RHD, or equally consistent with ARF/RHD). Or new diagnosis of RHD on echocardiogram (same echocardiogram findings). Evidence of inflammation on baseline laboratories (elevated ESR and/or CRP).
Known alternate diagnosis, overlapping	Clinical, laboratory or imaging data that provides sound evidence of an alternate diagnosis.
Clinical history of ARF or RHD but normal echocardiogram	Known history of prior ARF or RHD, but echocardiogram normal at enrolment.
Known alternate diagnosis, ASPGN with concern for concurrent ARF or RHD	Clinical and laboratory data that provides definitive evidence of an alternate diagnosis of ASPGN. Normal echocardiogram.
Known alternate diagnosis, ASPGN with concurrent likely ARF or RHD	Clinical and laboratory data that provides definitive evidence of an alternate diagnosis of ASPGN with echocardiogram features of ARF or RHD or clinical features of ARF.
Known alternate diagnosis, superficial GAS infection	Laboratory and clinical data that provides definitive evidence of an alternate diagnosis of acute GAS pharyngitis (confirmed by POC or microbiological culture (pharyngitis)). Or impetigo (photo) or scarlet fever (confirmed by POC or microbiological culture and photo of rash and/or tongue).
Healthy control	No evidence of acute inflammation or infectious conditions in the last 14 days based on clinical history and baseline laboratory testing (ESR <30 and CRP <3).
Possible ARF, suspected first case, likely new case	Streptococcal evidence, one major criteria+one minor criteria, but high suspicion (typically in the setting of normal inflammatory markers and pre-diagnostic NSAID use).
Unknown alternate diagnosis	Does not meet criteria for possible or definite ARF or healthy, but the case lacks definitive laboratory or imaging data to support a known alternate diagnosis.
Not enough evidence	Temporary category, asking sites for additional information, will be reviewed for further categorisation.

ARD, acute rheumatic fever; ASPGN, acute post-streptococcal glomerulonephritis; CRP, C-reactive protein; ESR, erythrocyte sedimentation rate; GAS, group A *Streptococcus*; NSAID, Nonsteroidal anti-inflammatory drugs; POC, Point of Care; RHD, rheumatic heart disease.

#### Patient and public involvement

Patients and members of the public were not involved in the design of this standardised multicentre recruitment protocol. The ARC Network protocol was developed to ensure harmonised case definitions, adjudication procedures and biospecimen collection across geographically and culturally diverse contexts. Local investigators and clinical teams at each recruitment site contributed contextual expertise to adapt recruitment materials, consent processes and community outreach strategies to local languages, health literacy levels and care-seeking behaviours.

## Discussion

ARF remains a persistent challenge in global cardiovascular health, particularly in low-resource settings where misdiagnosis or delayed recognition contributes to preventable morbidity and mortality.^21^,^23^ Even with access to comprehensive clinical and laboratory resources, diagnosis is complicated by the disease’s heterogeneous presentation and reliance on complex criteria. Recognising these barriers, the ARC Network has undertaken the most rigorous case adjudication to date and developed a series of diagnostic classifications that can serve as standardised tools for future ARF research. To further reduce variability, all participating sites receive standardised blood collection and biobanking kits, ensuring consistent sample quality and protocol fidelity. A critical limitation persists, however: the absence of a commercially available ADB ELISA. While our network is actively working to recreate this assay, its unavailability necessitates centralised testing in high-income countries and remains a global barrier to accurate ARF diagnosis.

The ARC framework integrates interdisciplinary collaboration with locally embedded expertise, aligning with contemporary priorities in biomarker development. Centralised resources in echocardiography, biobanking and adjudication complement strong local leadership, fostering data quality, protocol adherence and biospecimen integrity. Equity and local applicability are fundamental to the Network’s approach: by situating research within endemic communities and empowering local investigators, ARC ensures that scientific advances are culturally informed, clinically relevant and implementable. Building on this foundation, the Network is positioned to evolve into an enduring ARF research platform that spans diagnostic development, field testing and capacity building for future international therapeutics trials.

Annual face-to-face meetings are pivotal to this vision. They strengthen collaboration, facilitate knowledge exchange and promote alignment across sites. Beyond technical discussions, these gatherings cultivate trust and a shared sense of purpose, reinforcing the cohesion necessary for high-quality, coordinated research in diverse settings.

A highly sensitive and specific diagnostic test for ARF, designed for deployment in low-resource, RHD-endemic settings, remains a critical global priority. Such a tool would enable timely detection, appropriate treatment and prevention of long-term morbidity, addressing a gap that has long limited progress in ARF control. Equally important, advancing our understanding of ARF pathogenesis is essential for identifying therapeutics capable of altering disease progression and improving outcomes worldwide. The ARC Network is uniquely positioned to address both these imperatives: by combining rigorous case adjudication, standardised specimen collection and locally embedded research capacity with interdisciplinary expertise. In doing so, ARC aims not only to improve care in the communities it serves but also to provide a replicable framework for advancing ARF research globally, ultimately moving the field toward interventions that can meaningfully change the trajectory of this devastating disease.

## Dissemination of findings

Results from the ARC Network will be disseminated through peer-reviewed publications and presentations at scientific conferences. Recruitment sites receive regular feedback on study progress and key results to support local clinical practice and research capacity. Where appropriate, findings will also be shared with local clinicians, stakeholders and public health partners.

## Ethics statement

### Regulatory approval

A central Institutional Review Board (IRB) protocol was developed by the Clinical Coordinating Center and approved by the Cincinnati Children’s Hospital Medical Center IRB. The protocol includes customisable sections—such as recruitment strategies, consent procedures, participant retention plans and compensation details—to be tailored to the specific context of each recruitment site. Each site adapts these components as needed and obtains local ethical approval from their respective regulatory bodies prior to initiating participant recruitment. Prior to each enrolment, a parent or guardian signs a written informed consent and qualifying children, based on local age of assent, also sign written informed assent.

Recruitment sites;

Brazil: Universidade Estadual de Feira de Santana,

Approval number 6.636.220

Malawi: National Health Sciences Research Committee,

Approval number 3190

Timor-Leste: Institute National of Public Health Timor-Leste Research Ethics and Technical Committee,

Approval number 39INSP-TL/UEPD

Pakistan: University of Child Health Science, the Children’s Hospital Lahore Institutional Review Board,

Approval number 599

Biobank

The Kids Institute: Bellberry Human Research Ethics Committee,

Approval number 2023–04-425

Coordinating Center (prepared master protocol)

Cincinnati Children’s Hospital Institutional Review Board,

Approval number 2022–0946

### Oversight of biobank and clinical cohort data

The main purpose of this clinical recruitment is for discovery and validation of a diagnostic biomarker for ARF. Additionally, the ARC Network aims to improve our understanding of ARF pathogenesis and may also investigate prognostic ARC biomarker and novel ARF therapeutic targets. Use of biobank samples from other research teams for the purposes of ARF/RHD research will also be considered. All use of clinical data and samples is overseen by the ARC Network Core leadership team. Outside requests should be sent to: ARCdiagnosticnetwork@cchmc.org.

## Supplementary material

10.1136/bmjopen-2025-114968 online supplemental file 1
