# Supplementary material for: Acute Rheumatic Fever Diagnostic Network (ARC Network) clinical recruitment protocol
Source: BMJ Open. 2026 Mar 25;16(3):e114968. doi: 10.1136/bmjopen-2025-114968 (PMC13034315; doi:10.1136/bmjopen-2025-114968)
Supplement: online supplemental file 1 [file bmjopen-16-3-s001.docx]

**Supplemental Table**

Table S1: The ARC Network Investigators

| **Clinical Coordinating Center-Cincinnati Children’s Hospital (CCHMC)** | | | |
| --- | --- | --- | --- |
| **Name** | **Role** | **Institution** | **Country** |
| Andrea Beaton | Network Director | Cincinnati Children’s | USA |
| Samantha Buonfiglio | Network Coordinator | Cincinnati Children’s | USA |
| Ndate Fall | Scientific Coordinator | Cincinnati Children’s | USA |
| Robin Miller | Financial Coordinator | Cincinnati Children’s | USA |
| Rachel Sarnacki | Program Management | Cincinnati Children’s | USA |
| Nanhua Zhang | Biostatistician | Cincinnati Children’s | USA |
| **Recruitment Sites** | | | |
| Leticia Ferreira de Souza | Clinical Coordinator | Feira de Santana Children’s Hospital | Brazil |
| Isabella Fonseca | Fellow | Federal University of Minas Gerais | Brazil |
| Bernado Mendoza | Investigator | Feira de Santana Children’s Hospital | Brazil |
| Renata Fonseca Mendoza | PI | Federal University of Minas Gerais | Brazil |
| Maria Carmo P Nunes | PI | Federal University of Minas Gerais | Brazil |
| Sarah Gunter | Investigator | Baylor Foundation-Texas Children’s | Malawi |
| Norbert Kamchembere | Investigator | Kamuzu Central Hospital | Malawi |
| Manley Kamija | Investigator | University of North Carolina Project | Malawi |
| Mercy Kumwenda | Investigator | Baylor Foundation-Texas Children’s | Malawi |
| Treasure Mkaliainga | Investigator | Kamuzu Central Hospital | Malawi |
| Amy Sanyahumbi | PI | Baylor Foundation-Texas Children’s | Malawi |
| Morgan T. Sekou | Study staff | Baylor Foundation-Texas Children’s | Malawi |
| Tayamika Tambala | Clinical Coordinator | Baylor Foundation-Texas Children’s | Malawi |
| Omeir Aziz | Investigator | The Children’s Hospital, Lahore | Pakistan |
| Humera Javed | Investigator | The Children’s Hospital, Lahore | Pakistan |
| Tehmina Kazmi | Investigator | The Children’s Hospital, Lahore | Pakistan |
| Ansar Nawaz | Clinical Coordinator | The Children’s Hospital, Lahore | Pakistan |
| Masood Sadiq | PI | The Children’s Hospital, Lahore | Pakistan |
| Shahzadi Sardar | Study staff | The Children’s Hospital, Lahore | Pakistan |
| Meghan Bailey | Program Management | Menzies School of Health Research | Timor Leste |
| Sonia Freitas Belo | Study staff | Menzies School of Health Research | Timor Leste |
| Marciana da Costa | Study staff | Menzies School of Health Research | Timor Leste |
| Anferida Fernandes | Investigator | Menzies School of Health Research | Timor Leste |
| Aurora Fernandes | Study staff | Menzies School of Health Research | Timor Leste |
| Joshua Francis | PI | Menzies School of Health Research | Timor Leste |
| Armalino Gonzaga | Study staff | Menzies School of Health Research | Timor Leste |
| Merita Monteiro | Study staff | Menzies School of Health Research | Timor Leste |
| Herculano Seixas Dos Santos | Investigator | Menzies School of Health Research | Timor Leste |
| Messias Soares | Study staff | Menzies School of Health Research | Timor Leste |
| Deonisia de Fatima Soares | Study staff | Menzies School of Health Research | Timor Leste |
| Maria Itta Tanesi | Clinical Coordinator | Menzies School of Health Research | Timor Leste |
| Jennifer Yan | Investigator | Menzies School of Health Research | Timor Leste |
| **Echocardiography Core** | | | |
| Maria Carmo P Nunes | Member | Federal University of Minas Gerais | Brazil |
| Joselyn Rwebembera | Member | Uganda Heart Institute | Uganda |
| Craig Sable | Core Lead | Ochsner Children’s Hospital | USA |
| Amy Sanyahmbi | Member | Baylor Foundation-Texas Children’s | Malawi |
| Alison Spaziani | Coordinator | Children’s National Hospital | USA |
| Alison Tompsett | Coordinator | Children’s National Hospital | USA |
| **Diagnostic Core** | | | |
| Asha Bowen | Core Lead | The Kids Institute | Australia |
| Emma Ndagire | Member | Uganda Heart Institute | Uganda |
| Tom Parks | Member | Imperial College London | UK |
| Anna Ralph | Member | Menzies School of Health Research | Australia |
| Rachel Webb | Member | University of Auckland | New Zealand |
| **Biobank** | | | |
| Jonathan Carapetis | PI | The Kids Institute | Australia |
| Christine Everest | Program Management | The Kids Institute | Australia |
| Christopher Gorman | Biobank Manager | The Kids Institute | Australia |
| Jessical Hillas | Biobank coordinator | The Kids Institute | Australia |
| Lilly McLellan | Biobank Staff | The Kids Institute | Australia |
| **Biomarker Discovery** | | | |
| Timothy Barnet | Investigator | The Kids Institute | Australia |
| Timo Lassmann | Investigator | The Kids Institute | Australia |
| Wenna Lee | Investigator | The Kids Institute | Australia |
| Luke Marshall | Investigator | Biotome | Australia |
| Soraya Leedham | Investigator | Biotome | Australia |
| Samuel Lundin | Investigator | Biotome | Australia |
| Paige Warburton | Investigator | Biotome | Australia |
| Casey Shannon | Investigator | University of British Columbia | Canada |
| Scott Tebbutt | Investigator | University of British Columbia | Australia |
| Lauren Carlton | Fellow | University of Auckland | New Zealand |
| Micole Moreland | PI | University of Auckland | New Zealand |
| Reuben McGregor | Investigator | University of Auckland | New Zealand |
| Natalie Lorenz | Study Staff | University of Auckland | New Zealand |
| Ciara Ramiah | Fellow | University of Auckland | New Zealand |
| Wendy Shaddick | Collaborator | Counties Manukau Health | New Zealand |
| Monica De Oliveira Campos | Fellow | Imperial College London | UK |
| Rosemary Ho | Fellow | Imperial College London | UK |
| Myrsini Kaforou | Investigator | Imperial College London | UK |
| Michael Levin | Investigator | Imperial College London | UK |
| Lara Oberski | Fellow | Imperial College London | UK |
| Tom Parks | PI | Imperial College London | UK |
| Shiranee Sriskandan | Investigator | Imperial College London | UK |
| Richard Pouw | Investigator | Sanquin Research | Netherlands |
| **Scientific Oversight Committee** | | | |
| Mark Davies | Member | University of Melbourne | Australia |
| Mark Engel | Member | University of Cape Town | South Africa |
| Elizabeth Hartland | Member | Hudson Institute of Medical Research | USA |
| Ganesan Karthikeyan | Member | All India Institute of Medical Sciences | India |
| Justine Mintern | Member | University of Melbourne | Australia |
| Emmy Okello | Member | Uganda Heart Institute | Uganda |
| Andrew Steer | Member | University of Melbourne | Australia |
| Chris Van Beneden | Member | No Affiliation | USA |
| Liesl Zuhlke | Member | University of Cape Town | South Africa |

Table S2: Subcategories of non-GAS known alternate diagnoses

| Category | Examples from cohort |
| --- | --- |
| Suspected Connective Tissue Disease or Vasculitis | Juvenile idiopathic arthritis, systemic lupus erythematous |
| Suspected or Confirmed Arboviral Disease | Dengue fever |
| Suspected or Confirmed Asthma | Asthma exacerbation |
| Suspected or Confirmed Cardiomyopathy | Cardiomyopathy |
| Suspected or Confirmed Congenital Heart Disease | Atrial septal defect |
| Suspected or Confirmed Kawasaki Disease | Kawasaki Disease |
| Suspected or Confirmed Malaria | Malaria, cerebral malaria |
| Suspected or Confirmed Other Hematologic/Oncologic Disease | Leukaemia, lymphoma |
| Suspected or Confirmed Pharyngitis/Throat Infections | Non-GAS pharyngitis |
| Suspected or Confirmed Respiratory Infections | Pneumonia |
| Suspected or Confirmed Septic Arthritis | Septic arthritis |
| Suspected or Confirmed Sickle Cell Disease | Sickle Cell Crisis |
| Suspected Pericarditis, Myopericarditis or Endocarditis | Infective endocarditis |
| Other Bacterial Infections | Cellulitis, pyelonephritis, bacteraemia |
| Other Infectious Diseases | Chagas disease |
| Other Neurologic Disease | Partial seizure |
| Other Renal Disease | Nephrotic syndrome |
| Other Skin Diseases | Erythema nodosum |
| Other Uncertain Valvular Disease | Non-rheumatic valvular heart disease |
