# Supplementary material for: Acute Rheumatic Fever Diagnostic Network (ARC Network) clinical recruitment protocol
Source: BMJ Open. 2026 Mar 25;16(3):e114968. doi: 10.1136/bmjopen-2025-114968 (PMC13034315; doi:10.1136/bmjopen-2025-114968)
Supplement: Supplementary file 2 [file bmjopen-2025-114968.reviewer_comments.pdf]

PEER REVIEW HISTORY

BMJ Open publishes all reviews undertaken for accepted manuscripts. Reviewers are asked to complete a checklist review form and are provided with free text boxes to elaborate on their assessment. These free text comments are reproduced below.

ARTICLE DETAILS

Title (Provisional)

The Acute Rheumatic Fever Diagnostic Network (ARC Network) Clinical Recruitment Protocol

Authors

Fall, Ndate; Bowen, Asha Clare; Buonfiglio, Samantha; Francis, Joshua Reginald; Gorman, Christopher; Fonseca Mendoza, Renata; Fernandes, Ela; Moreland, Nicole J; Pereira Nunes, Maria Carmo; Sable, Craig; Sadiq, Masood; Shannon, Casey P.; Parks, Tom; Ralph, Anna; Sanyahumbi, Amy; Sarnacki, Rachel; Tebbutt, Scott; Webb, Rachel; Carapetis, Jonathan; Beaton, Andrea Zawacki; ARC, Network

---

VERSION 1 - REVIEW

**Reviewer**	**1**
**Name**	**Femi-Lawal, Victor**
**Affiliation**	**University College Hospital Ibadan**
**Date**	**18-Jan-2026**
**COI**	**None**

Reviewer Comments

ABSTRACT

Some minor grammatical errors in the Introduction section.

In the Discussion, it is said ARF is difficult to diagnose in low-resource settings. But as you also highlighted in the Introduction, “no diagnostic test currently exists”. Thus, while it is largely correct that it is more difficult to diagnose ARF in low-resource settings, it may be better to state that ARF is difficult to diagnose in general (and then add an emphasis of the extra difficulty in LMICs).

Manuscript

Grammatical error. Absence of an article in line 9 of page 2. Additionally, patient involvement is spelled wrongly in the subheading.

Is there a role for local experts in the coordinating center or the scientific oversight committee? Please clarify.

In line 38-39 you mention the recruitment of children with superficial group A Streptococcus infections but no ARF. Considering that the development of ARF is a long-term sequela of superficial Strep pyogenes infection, could you clarify how/if you would separate the children who would eventually develop ARF from this group, to prevent confounding? Or is there a particular time period at which the children would be deemed not to have acquired ARF and thus certified clear? Please clarify.

Is there a reason why 250 participants is the target size? Kindly provide a brief justification (statistical, biomolecular, etc.) for the sample size.

In line 48 – 50, oral health is stated twice.

Add a brief note on the validation of the tools for assessing quality of life in the study settings (four study sites) as stated in lines 51-52.

In the manuscript, it would be vital to briefly state how key findings will be disseminated. If this has been stated in another publication, kindly cite that.

VERSION 1 - AUTHOR RESPONSE

Reviewer 1 Comments

We thank the reviewer for their careful reading and valuable suggestions.

ABSTRACT

1. Minor grammatical errors in the Introduction section.

Response:
We thank the reviewer for this helpful observation. The abstract has been carefully revised to correct minor grammatical errors in the Introduction section.

2. Suggest revising statement that ARF is difficult to diagnose in low-resource settings, given no diagnostic test exists.

Response:

Although the Discussion section has been removed from the Abstract in accordance with BMJ Open guidelines for protocol articles, we have revised the relevant language in the manuscript “Introduction” section (page 2) to clarify that ARF is inherently difficult to diagnose due to the absence of a definitive diagnostic test, with additional diagnostic challenges in low- and middle-income settings where access to laboratory and imaging resources may be limited. This revision ensures conceptual consistency between the Introduction and the main text. This change better reflects the global diagnostic challenge

while emphasizing inequities.

MANUSCRIPT COMMENTS

1. Grammatical error (absence of article, page 2 line 9) and spelling error in "patient involvement".

Response:
Both errors have been corrected in the revised manuscript. We have also performed a full proofreading of the document to address similar minor issues.

2. Is there a role for local experts in the Coordinating Center or Scientific Oversight Committee?

Response:
Thank you for highlighting this. We have clarified in the revised manuscript method section that:

- The Clinical Coordinating Center works closely with local site investigators, who play a central role in recruitment oversight and contextual protocol implementation (page 4).
- The Scientific Oversight Committee includes internationally recognized experts, including representation from ARF-endemic regions (page 5).

3. Superficial GAS group – how will you account for those who may later develop ARF?

Response:
Thank you for this important question. Participants enrolled in the Superficial GAS category are evaluated at the time of presentation and must not meet criteria for ARF at enrollment based on standardized clinical assessment, laboratory evaluation, echocardiography, and central adjudication. Diagnostic classification is determined contemporaneously using the ARC adjudication framework.

As this is a cross-sectional recruitment protocol designed to support biomarker discovery, participants are categorized based on their adjudicated diagnosis at enrollment. The biospecimens bio banked for downstream biomarker analyses are linked to this enrollment diagnosis, ensuring that discovery analyses reflect rigorously defined baseline phenotypes. Individuals who subsequently develop ARF would represent incident cases occurring after study classification and would not alter the validity of the original enrollment categorization.

We believe this approach is appropriately reflected in the current manuscript and does not require additional modification.

4. Justification for target sample size of 250 participants.

Response:
We appreciate this request for clarification. We have revised the manuscript to provide a more explicit methodological justification for the target sample size.

The ARC Network employs a phased biomarker discovery and validation strategy aligned with a PRoBE design (Prospective Specimen Collection with Retrospective Blinded Evaluation), which is recommended to improve the rigor and reproducibility of biomarker research (Pepe et al., 2015). In the initial recruitment phase, the target of ≥250 participants (≥50 per recruitment category) was selected to ensure adequate phenotypic representation across four endemic sites while maintaining feasibility and geographic diversity.

This recruitment phase contributes to a larger planned validation cohort (n≈1000; ~200 cases and ~800 controls), which is powered to support robust model development and independent validation. Based on established guidance for clinical prediction modeling and shrinkage considerations (Riley et al., 2021), this sample size allows:

- Limited risk of overfitting (<10% coefficient shrinkage),
- Precise estimation of model discrimination (±0.05), and
- Evaluation of models of increasing complexity (AUC ≥0.80–0.90 with up to 20–40 parameters).

This clarification has been added to the Recruitment Categories section (page 6) to better articulate the statistical and methodological rationale underpinning the enrollment targets.

5. Oral health listed twice (line 48–50).

Response:
Thank you. The duplication has been corrected.

6. Add note on validation of quality-of-life tools across sites.

Response:
We appreciate this important suggestion. We have revised the Methods section to clarify that the PedsQL Generic Core Scales were selected due to their extensive cross-cultural validation. The instrument has been translated into more than 60 languages and has demonstrated reliable and valid psychometric properties across diverse international pediatric populations (Varni et al., 2005). We now also explicitly note that local validation was not conducted within the four participating study countries, and this will be acknowledged as a limitation in resulting publications. This clarification has been incorporated into the Methods section under the Risk Factor paragraph (page 7).

7. Clarify how key findings will be disseminated.

Response:
We have expanded the Ethics and Dissemination section to include:

- Dissemination of findings through peer-reviewed publications and conference presentations
- Feedback to participating sites
- Sharing of findings with local clinicians and stakeholders

This addition strengthens transparency and aligns with protocol reporting standards and has been added to the Ethics and Dissemination section (page 11)
